# Transcriptional Networks in *S. cerevisiae* Linked to an Accumulation of Base Excision Repair Intermediates

**DOI:** 10.1371/journal.pone.0001252

**Published:** 2007-11-28

**Authors:** Ivan Rusyn, Rebecca C. Fry, Thomas J. Begley, Joanna Klapacz, J. Peter Svensson, Mark Ambrose, Leona D. Samson

**Affiliations:** 1 Center for Environmental Health Sciences and Department of Biological Engineering, Massachusetts Institute of Technology, Cambridge, Massachusetts, United States of America; Pasteur Institute, France

## Abstract

Upon exposure to DNA damaging agents, *Saccharomyces cerevisiae* respond by activating a massive transcriptional program that reflects the fact that “DNA damaging” agents also damage other cellular macromolecules. To identify the transcriptional response that is specific to DNA damage, we have modulated the first two enzymes in the base excision repair (BER) pathway generating yeast strains with varied levels of the repair intermediates, abasic sites or strand breaks. We show that the number of abasic sites is significantly increased when the 3-methyladenine DNA glycosylase (Mag): AP endonuclease (Apn1) ratio is increased and that spontaneous frame shift mutation is considerably elevated when either Mag, or Mag plus Apn1, expression is elevated. Expression profiling identified 633 ORFs with differential expression associated with BER modulation. Analysis of transcriptional networks associated with the accumulation of DNA repair intermediates identifies an enrichment for numerous biological processes. Moreover, most of the BER-activated transcriptional response was independent of the classical yeast environmental stress response (ESR). This study highlights that DNA damage in the form of abasic sites or strand breaks resulting from BER modulation is a trigger for substantial genome-wide change and that this response is largely ESR-independent. Taken together, these results suggest that a branch point exists in the current model for DNA damage-signaled transcription in *S. cerevisiae*.

## Introduction

Previously, we have shown that yeast exposed to alkylating agents initiate a broad gene expression response modulating a staggering 30% of the yeast genome, i.e. up to 2000 genes [1], [2]. Upon exposure to exogenous alkylation damage, yeast respond by modulating a host of biological processes that includes, as expected, DNA repair and cell cycle checkpoint pathways, but also includes the following: (i) activation of protein degradation machinery, (ii) increased lipid metabolism, (iii) increased RNA processing and (iv) decreased protein synthesis (ribosomal biosynthesis) [1], [2]. That numerous biological processes are modulated when yeast are exposed to alkylating agents likely reflects the fact that in addition to damaging DNA, these so-called “DNA damaging” agents also damage proteins, lipids and RNA. In addition, the expression of a core set of ∼900 genes changes under a variety of stressful conditions, including DNA damage, and is termed the environmental stress response (ESR) [3]. Here, we set out to identify the transcriptional response that is specifically caused by damage to DNA and not to other cellular molecules; instead of introducing damage with reactive electrophilic agents (e.g. an alkylating agent) we generated DNA damage by imbalancing the base excision repair (BER) pathway such that abasic sites or gaps and nicks accumulate in the genome during the processing of repairing endogenous DNA damage.

In its simplest form, the first two steps of the BER pathway are represented by a DNA glycosylase and an AP endonuclease. DNA glycosylases can recognize damaged bases and remove them via cleavage of the *N*-glycosidic bond to initiate BER. In *S. cerevisiae* there are at least five distinct DNA glycosylases, namely Ung, Nth1, Nth2, Ogg1, and Mag, that collectively recognize damaged bases and process them to generate abasic sites. The abasic site is substrate for AP endonucleases that carry out the next step in BER. *S. cerevisiae* has two distinct AP endonucleases, the major enzyme Apn1 and a damage-inducible enzyme, Apn2 [4]. Apn1 and Apn2 cleave abasic sites 5′ to the site of base loss, generating a single strand break. The abasic sugar residue is removed by deoxyribophophodiesterase, the gap is filled by DNA polymerase and the remaining nick sealed by DNA ligase.

Across phylogeny, the regulation of enzymes involved in the first two steps of BER has been noted at the level of transcription. In *E. coli*, yeast, mouse and human cells, treatments with xenobiotics can induce the transcription of BER genes [5]–[10]. The induced expression of such DNA repair pathways has long been thought to confer protection against both the killing and mutagenic effects of DNA damage [11]. However, it is now apparent that in some circumstances increased expression of DNA repair genes may actually lead to the accumulation of mutations. In fact, our previous studies demonstrated the generation of a strong mutator phenotype in *S. cerevisiae* and *E. coli* by imbalanced base excision repair [12]–[15]. Specifically, it was shown that Mag expression in yeast produces up to a 300-fold increase in the spontaneous base substitution mutation rate when Mag levels are high relative to Apn1 [12]. It was suggested that such a dramatic increase in spontaneous mutation rate is due to the fact that Mag excises normal DNA bases, in addition to endogenously damaged bases, producing mutagenic AP sites that can be further processed into mutations by the *Rev1/Rev3/Rev7* lesion bypass polymerases [12], [15], [16].

In these studies, we explore how imbalanced BER affects the persistence of AP sites and frameshift mutations in yeast and how the accumulation of BER intermediates is linked to a DNA damage specific transcriptional response. We show that Mag expression in *S. cerevisiae* produces a 2- to 5-fold increase in AP sites, depending on the Mag:Apn1 ratio, an effect that mirrors the changes in spontaneous base substitution mutation rates in the same strains of yeast [12]. This finding confirms the hypothesis that increased Mag:Apn1 ratios lead to an increased steady state level of mutagenic AP sites. Furthermore, frameshift mutations are elevated when Mag:Apn1 ratios are high and these mutations are reduced with increased expression of Apn1. These studies also show that in response to the DNA damage of accumulated BER repair intermediates, yeast respond by modulating the expression of ∼600 ORFs that are integrated into transcriptional networks encoding DNA replication/metabolism, protein transport, energy metabolism and ribosomal biosynthesis machinery. Surprisingly, this BER-modulated transcriptional response is largely independent of the environmental stress response (ESR), suggesting that a branch point exists in the current model for DNA damage signaled transcription.

## Results

### Imbalance in the first two steps in BER leads to accumulation of AP sites

**Figure 1:**
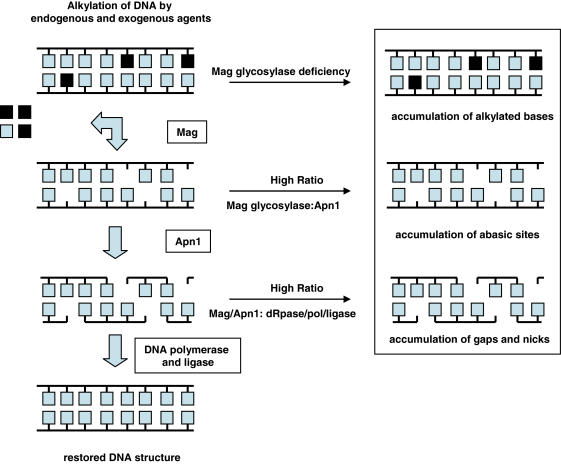
Imbalances of base excision repair. The pathway on the left indicates normal processing in the base excision repair pathway. Central arrows highlight the potential imbalances that may lead to toxicity, clastogenecity and mutagenicity on the right (modified from [26]).

In this study, the first two enzymes in the BER pathway, Mag and Apn1, were modulated such that the BER intermediates, abasic sites or strand breaks, accumulate (Fig. 1). Specifically, the impact of accumulating abasic sites was examined by (i) increased expression of Mag while maintaining wild type levels of Apn1 (WT/p*MAG*), (ii) maintaining wild type levels of Mag in an Apn1 deficient strain (Δ*apn1*), or (iii) increased expression of Mag in an Apn1 deficient strain (Δ*apn1*/p*MAG*). The impact of accumulating gaps and nicks was examined by (i) expressing Apn1 (Δ*apn1*/p*APN1*), or (ii) expressing both Mag and Apn1 (Δ*apn1*/p*MAG*/p*APN1*) (Table 1).

**Table 1:** Yeast strains used in this study.

Strain	Genotype	Experimental Group
BGY111	*MATa his3 leu2 ura3-52 trp1-289_a_ can1* GAL+	Parent
BGY116	BGY111/pYES	Wild Type
BGY119	BGY111/pYES-MAG	WT*/*p*MAG*
BGY122	BGY111 *apn1-1::HIS3*/pYES	*Δapn1*
BGY125	BGY111 *apn1-1::HIS3*/pYES-MAG	*Δapn1/*p*MAG*
BGY195	BGY111 *apn1-1::HIS3/*pYepAPN1-2A/pYES	*Δapn1/*p*APN1*
BGY196	BGY111 *apn1-1::HIS3/*pYepAPN1-2A/pYES-MAG	*Δapn1/*p*MAG/*p*APN1*

**Figure 2:**
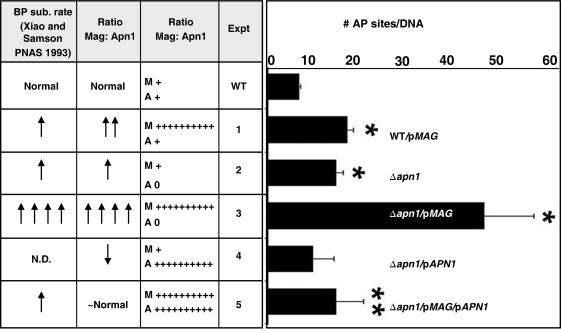
Five BER modulated strains were compared for measurements of AP site accumulation. Five experimental conditions (#1–5) each with different ratios of Mag:Apn1 were assessed for AP site accumulation measured in genomic DNA. Statistical difference (t-test, p<0.05) from wild type (WT, *) or from Δ*apn1/MAG* group (**) is indicated. The expression of Mag of Apn1 are shown as: (0) = low expression, (+) = wild type expression, (++++++++++) = high expression.

The persistence of AP sites in the yeast strains with altered levels of expression of Apn1 and Mag was examined. Plasmid based Mag expression in the wild type host increased steady-state level of genomic AP sites by ∼2.5 fold (Fig. 2, Expt. #1). In the Δ*apn1* strain, AP sites increased ∼2-fold, an effect that was essentially reversed by expressing Apn1 from a plasmid (Fig. 2, Expt. #2, #4). As one might expect, when Mag was expressed in the Δ*apn1* strain, AP sites were further elevated to ∼5-fold that in wild type cells (Fig. 2, Expt. #3). When Apn1 was coexpressed with Mag in the Δ*apn1* strain, the steady state level of AP sites fell dramatically, but it should be noted that it did not quite return to wild type levels (Fig. 2, Expt. #5).

### Frame shift mutations accumulate under conditions of increased abasic sites and increased gaps and nicks

**Figure 3:**
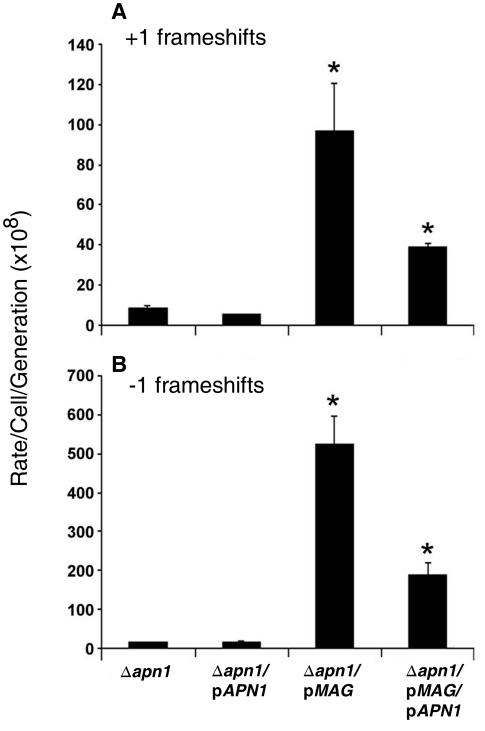
Induction of base excision repair leads to an accumulation of frameshift mutations. Frameshift mutation frequency in Δ*apn1* strains that expressed Apn1 and/or Mag was determined as detailed in Materials and Methods. Asterisk (*) denotes statistical difference (t-test, p<0.05) relative to the background strain (Δ*apn1*).

In addition to the AP site measurements in the BER modulated strain, we set out to examine another common type of spontaneous-induced damage, namely (+1) and (−1) frame shift mutations. We find that Apn1 expression does not affect frame shift mutation rates (Fig. 3). However, Mag expression caused a dramatic increase of 12 and 38-fold in (+1) and (−1) frame shifts respectively when compared to the Apn1-deficient strain. Interestingly, co-expression of Apn1 and Mag did not fully suppress the increased frame shift mutagenesis with a remarkable 5- and 14-fold increase in (+1) and (−1) frame shifts, respectively, still remaining (Fig. 3).

### Accumulation of BER intermediates results in DNA damage specific genome-wide signatures distinct from signatures induced by MMS

**Figure 4:**
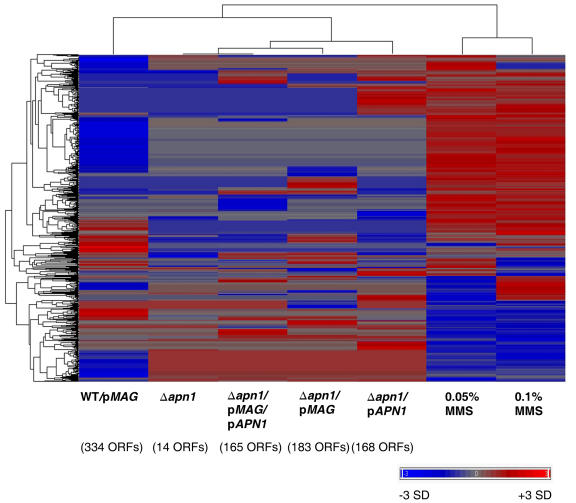
Gene expression changes in *S. cerevisiae* caused by an induction of base excision repair or methyl methane sulfonate (MMS). MMS transcriptional data were obtained from two doses (0.05% and 0.1%) from [1]. Unsupervised hierarchical clustering of 633 ORFs whose transcription was significantly changed in at least one of the BER strains used in this study (as compared to the wild type). The number of ORFs identified for each experiment as statistically significant is shown in brackets below the column. Fold changes were Z-score transformed; red indicates increased relative expression, and blue indicates decreased relative expression.

The genome-wide response of yeast to the accumulation of DNA repair intermediates was examined by performing transcriptional profiling of the five yeast strains with altered expression of BER repair enzymes (see Materials and Methods). The genome-wide expression levels of the BER modulated strains were compared to wild type yeast and the differentially expressed genes were identified (see Materials and Methods); a total of 633 ORFs were identified with statistically significant changes compared to wild type yeast in at least one condition (Fig. 4, Dataset S1). It should be emphasized here that these strains were not exposed to any exogenous DNA damaging agents. The extent of change across the yeast genome in response to BER expression was quite varied across the five experiments, with yeast expressing Mag (WT/p*MAG*) showing the most transcriptional change, specifically 334 modulated ORFs (Fig. 4).

We further set out to compare the transcriptional response that was specifically caused by the accumulation of DNA repair intermediates, to that caused by exposure to an alkylating agent. The expression patterns of the BER modulated strains were compared to our previously published data of the transcriptional response of yeast when exposed to the alkylating agent, methyl methanesulfonate (MMS), at two doses 0.05% and 0.1% [1]. The expression patterns of the five yeast strains with modulated levels of BER were strikingly different from the expression patterns of yeast exposed to MMS (Fig. 4).

### The accumulation of BER intermediates activates ESR-independent transcriptional networks enriched for numerous biological processes

To identify whether significant molecular interactions exist among the gene products of the transcripts that were modulated by BER intermediates, the 633 ORFs that were transcriptionally modulated in at least one BER condition were overlayed on a functional yeast network comprised of 4,691 nodes (proteins) and 34,000 edges (interactions) [17]. Of the 633 ORFs modulated by expressing BER enzymes, 501 were contained in the large yeast network (Fig. S1, Dataset S1).

**Figure 5:**
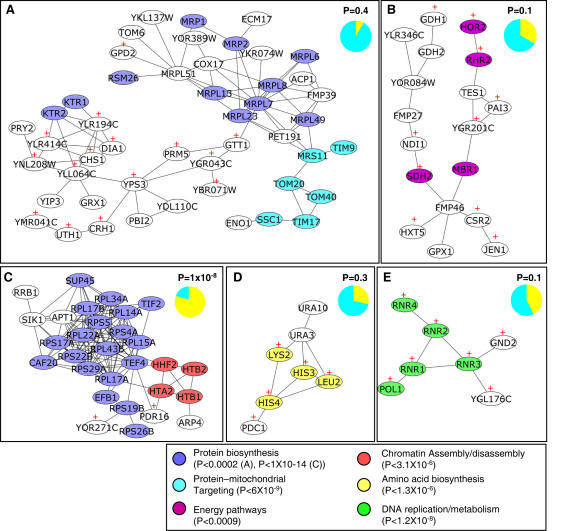
Yeast transcriptional networks activated in response to BER intermediate accumulation are largely ESR-independent. A) Subnetwork #1 enriched for protein-mitochondrial targeting and protein biosynthesis, B) Subnetwork #2 enriched for energy pathways, C) Subnetwork #3 enriched for ribosome biogenesis and chromatin assembly, D) Subnetwork #4 enriched for amino acid biosynthesis, E) Subnetwork #5 enriched for DNA replication/metabolism. P-values for gene ontology enrichment are indicated in legend. Pie charts in the upper right hand corner of each figure represent the percentage of network nodes that are ESR (yellow) or non ESR (blue) (P-value of ESR enrichment is shown). Proteins encoded by transcripts that are transcriptionally activated are indicated with a (+), all other proteins are encoded by transcripts that are transcriptionally repressed by BER intermediate accumulation.

To identify transcriptionally enriched modules within the protein network, we further analyzed this large interactome for clusters of proteins that interact and also are modulated by BER intermediate accumulation; five subnetworks were identified (Fig. S1). It should be mentioned again that these subnetworks are encoded entirely by genes that are transcriptionally modulated by BER intermediate accumulation. The subnetworks that are modulated by BER intermediate accumulation are enriched for numerous biological processes including: protein biosynthesis (Fig. 5A, C), protein-mitochondrial targeting (Fig. 5A), energy pathways (Fig. 5B), chromatin assembly/disassembly (Fig. 5C), amino acid biosynthesis (Fig. 5D), and DNA replication/metabolism (Fig. 5E). Of these biological processes that are modulated by BER intermediate accumulation, those that are transcriptionally activated are: (i) amino acid biosynthesis, (ii) DNA replication/metabolism, and (iii) energy pathways (Fig. 5). The biological processes that are down regulated by BER intermediate accumulation are protein biosynthesis, chromatin assembly/disassembly and protein-mitochondrial targeting (Fig. 5).

### The DNA damage-specific response to BER intermediates is largely ESR independent

The current model of DNA damage-induced transcriptional response in yeast suggests that DNA damage signals for the modulation of three groups of genes; (i) the activation or repression of a battery of ∼900 genes classified as the environmental stress response (ESR), (ii) the modulation of ∼50 cell cycle associated genes, and (iii) the modulation of 9 genes termed the “DNA damage signature” [18]. We set out to determine whether or not the transcriptional networks that were activated by the accumulation of BER intermediates were largely comprised of the ESR, as would be predicted by the current model. The statistical enrichment of ESR genes was calculated for each of the subnetworks, as well as for the entire list of 633 ORFs modulated by BER intermediate accumulation (see Materials and Methods) (Fig. 5 A–E). Surprisingly, the enrichment of the ESR in the set of 633 ORFs was not statistically significant (P = 0.12). Furthermore, only one of the five subnetworks was significantly enriched for genes that belong to the ESR (Fig. 5 C). The third subnetwork is highly enriched for proteins that are involved in ribosome biogenesis; note that a classic hallmark of the ESR is that ribosomal protein genes are repressed (Fig. 5C) [18]. All of the other subnetworks that were activated by the accumulation of BER intermediates were statistically ESR-independent.

It should be mentioned that contained within these BER intermediate modulated transcriptional networks are a few members of the “DNA damage signature” and transcripts that encode members of the cell-cycle related genes. Specifically, the fifth subnetwork that is enriched for DNA replication/metabolism integrates two members of the yeast “DNA damage signature,” namely the ribonucleotide reductases Rnr2 and Rnr4. The third subnetwork that is enriched for chromatin assembly/disassembly-related proteins contains two cell-cycle related transcripts that encode histones, Htb1 and Htb2, found previously to be repressed by MMS and IR [18].

**Figure 6:**
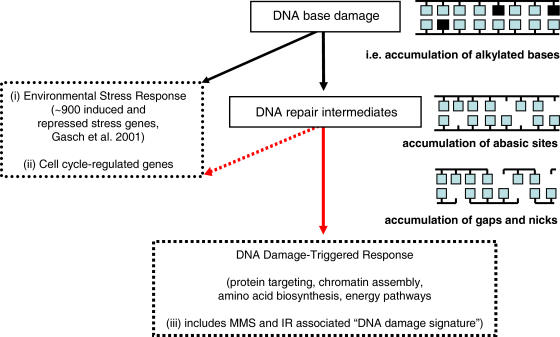
Diagram of a DNA damage-triggered transcriptional response in *S. cerevisiae*. DNA base damage (e.g. alkylated bases induced by endogenous or exogenous agents) is processed by repair machinery resulting in repair intermediates (e.g. abasic sites, gaps and nicks). DNA damage signals for the transcriptional modulation of (i) the induction or repression of the ∼900 genes belonging to the environmental stress response (ESR), and (ii) ∼50 cell cycle regulated genes, and (iii) DNA damage-triggered networks that include the MMS and IR DNA damage signature. Novel branch points in the DNA damage-triggered ESR independent transcriptional response are shown in red.

Our network findings suggest that the current model of DNA damage-induced transcriptional activation in yeast should be expanded in a few ways (Fig. 6). First, our results show that DNA damage in the form of repair intermediates is insufficient to activate the bulk of the environmental stress response. Second, only a subset of the MMS and IR “DNA damage signature” is activated by the accumulation of repair intermediates. As the DNA damage-specific response to BER intermediates is largely ESR independent, these data suggest that a branch point in the current model of DNA damage-induced transcriptional signaling exists (Fig. 6). Specifically, we find transcripts that encode proteins involved in protein-mitochondial targeting, chromatin assembly/disassembly, DNA replication/metabolism and energy pathways, are members of the DNA damage activated gene set.

## Discussion

In this study we set out to identify the transcriptional response in yeast that is specifically caused by damage to DNA and not to other cellular molecules. As an alternative to introducing damage with reactive electrophilic agents (e.g. an alkylating agent), we generated DNA damage by imbalancing the base excision repair (BER) pathway such that abasic sites or gaps and nicks accumulate in the genome during the process of repairing endogenous DNA damage. Five yeast strains with altered expression levels of Apn1 and Mag were analyzed for the persistence of AP sites. Mag expression in the wild type host increased the steady-state level of genomic AP sites by ∼2.5 fold. As expected, when Mag was expressed in the Δ*apn1* strain, AP sites were further elevated to ∼5-fold that in wild type, whereas coexpression of Apn1 with Mag in the Δ*apn1* strain resulted in a dramatic decrease in the steady state level of AP sites. These results complement our findings for single base substitution mutation rates in the same strains of yeast where an increase in Mag: Apn1 ratio leads to the generation of a strong mutator phenotype in yeast [12]. It was found that spontaneous mutations were increased several hundred fold when Mag expression was relatively high and Apn1 relatively low [12], [15]. Intriguingly, while Mag expression in AP endonuclease-deficient yeast led to a nearly 300-fold increase in spontaneous mutation rate, here we observed only a 5-fold increase in steady state levels of AP sites. However, unlike the current experiments with logarithmically growing cells, spontaneous mutation rates were determined in stationary phase cells over a period of 14 days [12], [15]. Since stationary phase yeast cells are known to exhibit significantly higher levels of oxidant production [19], the difference in experimental conditions might account for this apparent divergence. Indeed, when AP sites were measured in 7 day old stationary phase cells, a further >10-fold increase in AP sites was detected in wild type and Mag expressing strains (data not shown). We further examined the BER modulated yeast strains for another type of spontaneous-induced damage, that of frame shift mutations. Similar to our results with spontaneous base substitution rates [12], [15], we find that Apn1 expression does not affect frame shift mutation rates. Mag expression, however, caused a dramatic increase in frame shift mutation rate when compared to the Apn1-deficient strain.

To determine the impact of the accumulation of DNA repair intermediates on gene expression, the five yeast strains with altered expression of BER repair enzymes were analyzed for differential gene expression. A total of 633 ORFs were identified with statistically significant changes in at least one condition compared to wild type yeast. The extent of change across the yeast genome in response to modulated BER expression was quite varied across the five experiments, with yeast expressing Mag (WT/p*MAG*) showing the most transcriptional change. It is noteworthy that even without exposure to any exogenous DNA damaging agents there is considerable gene expression change.

The genome-wide impact of the accumulation of DNA repair intermediates was compared to our previous results of gene expression changes in yeast upon exposure to an alkylating agent, namely methyl methanesulfonate (MMS) [1]. It was a surprise to find the expression patterns of the five yeast strains with modulated levels of BER were strikingly different from the expression patterns of yeast exposed to MMS. These differences in the expression patterns of the BER modulated yeast strains and yeast exposed to MMS may, in part, represent the difference between responses to an acute influx of damage, as for MMS, rather than a chronic exposure to damage, as would be generated by BER modulation.

To classify the biological processes and pathways that were affected by BER intermediate accumulation, the gene products of the transcripts that were modulated by BER intermediates, were overlayed on a functional yeast network [17]. A large interacting networks of proteins was identified that contained five transcriptionally enriched subnetworks. These subnetworks are encoded entirely by genes that are transcriptionally modulated by BER intermediate accumulation. These subnetworks are enriched for numerous biological processes including: protein biosynthesis, protein-mitochondrial targeting, energy pathways, chromatin assembly/disassembly, amino acid biosynthesis and DNA replication/metabolism. Three of these biological processes that are modulated by BER intermediate accumulation are transcriptionally activated; these are amino acid biosynthesis, DNA replication/metabolism, and energy pathways. The protein biosynthesis and protein-mitochondrial targeting processes are down regulated by BER intermediate accumulation.

The panel of biological processes that in this study are modulated by BER intermediate accumulation is strikingly absent of a number of cellular processes previously shown to be modulated in yeast exposed to the alkylating agent MMS [1], [2]. Notably missing from the list of biological processes modulated by endogenous DNA damage are (i) the 26S proteasome, (ii) lipid metabolism machinery, and (iii) RNA processing machinery. That these processes are modulated when yeast strains are exposed to exogenously generated alkylation damage likely represents the non-specific nature of the damage (e.g. damage to proteins, lipids and RNA) induced by alkylating agents.

To date, the proposed model of DNA damage-induced transcriptional response in yeast suggests that DNA damage signals for the modulation of ∼900 genes classified as the environmental stress response (ESR), but also the modulation of ∼50 cell cycle associated genes, and 9 genes termed the “DNA damage signature” [18]. In addition to their response to DNA damaging agents, the ESR genes in yeast are also responsive to a host of changes in environmental conditions [3]. We set out to determine whether or not the transcriptional networks that were activated by the accumulation of BER intermediates were largely comprised of the ESR, as would be predicted by the current model of DNA damage-induced transcriptional response. The statistical enrichment of ESR genes was calculated for each of the subnetworks, as well as for the entire list of ORFs modulated by BER intermediate accumulation in the yeast strains. Surprisingly, the enrichment of the ESR in the set of 633 ORFs was not statistically significant. Furthermore, only one of the five subnetworks was significantly enriched for genes that belong to the ESR. All of the other subnetworks that were activated by the accumulation of BER intermediates were statistically ESR-independent.

### Conclusions

Our results show that imbalancing the BER pathway leads to an accumulation of repair intermediates, such as AP sites, and stimulates the induction of frame shift mutations. Furthermore, we show that the induction of DNA repair intermediates, even in the absence of an exogenous damaging agent, causes a profound change in gene expression *S. cerevisiae*. This DNA damage-specific expression pattern of ∼600 ORFs varies markedly from the expression pattern produced by exogenous exposure to the alkyating agent MMS. The biological processes modulated by the presence of DNA repair intermediates include protein synthesis, protein targeting, amino acid biosynthesis, and chromatin assembly. Notably lacking from the biological processes modulated by DNA repair intermediates is that of the protein degradation machinery (e.g. 26S proteasome) which is robustly induced upon exposure to exogenous DNA damaging agents; this suggests that the trigger for upregulating the proteasome emanates from damaged molecules other than DNA, most likely from damaged proteins. The DNA damage-triggered transcripts encode molecular networks that are comprised of proteins involved, not surprisingly, in DNA replication/metabolism, but also in numerous other biological processes but that the triggered responses is largely ESR-independent. As the DNA damage-specific response to BER intermediates is largely ESR independent, these data suggest that a branch point in the current model of DNA damage-induced transcriptional signaling exists. This study highlights that DNA damage, specifically, is the trigger for the modulation of transcripts that encode proteins with broad biological functions.

## Materials and Methods

### Strains and plasmids

Table 1 lists the strains used in this study. All strains, plasmids and cell growth conditions were as previously described [12]. Briefly, strains were grown from overnights in SD-*ura*+GAL media at 30°C with constant shaking until cell density of 10^7^ cells/ml. They were then pelleted by centrifugation, quick-frozen using a dry ice/ethanol bath, and stored at −80°C for use in the assays described below. All experiments were performed in biological triplicate.

### Isolation of DNA

DNA was extracted by a procedure slightly modified from the method reported previously [20]. To minimize formation of oxidative artifacts during isolation, 2,2,6,6-tetramethylpiperidinoxyl (TEMPO, 20 mM final concentration) was added to all solutions and all procedures were performed on ice. Briefly, frozen cells were thawed and homogenized in PBS with a Tehran homogenizer (Wheaton Instruments, Millville, NJ). After centrifugation at 2,000×g for 10 min, the nuclear pellets were incubated in lysis buffer (Applied Biosystems) overnight at 4°C with proteinase K (500 mg/ml; Applied Biosystems). DNA was then extracted twice with a mixture of phenol/chloroform/water followed by ethanol precipitation. The extracted DNA was incubated in PBS (pH 7.4) with RNase A followed by DNA precipitation with cold ethanol. Then, the DNA pellet was resuspended in sterilized distilled water. The DNA solution was stored at −80°C until assayed.

### Abasic sites

Abasic (AP) sites were measured based on a procedure reported by Nakamura and Swenberg [21]. Briefly, 8 µg of DNA in 150 µl of phosphate-buffered saline was incubated with 1 mM aldehyde reactive probe at 37°C for 10 min. After precipitation using cold ethanol, DNA was suspended in TE buffer. DNA (250 ng) in TE buffer was heat-denatured and loaded on a nitrocellulose membrane (110 ng DNA/slot, Hybond-C Super, Amersham Pharmacia Biotech) and soaked with 5× SSC then baked in a vacuum oven for 30 min. The membrane was preincubated with 10 ml of Tris-HCl containing bovine serum albumin for 15 min and then incubated in the same solution containing streptavidin-conjugated horseradish peroxidase at room temperature for 45 min. After rinsing the nitrocellulose membrane, the enzymatic activity on the membrane was visualized by enhanced chemiluminescence reagents. The nitrocellulose filter was exposed to x-ray film, and the developed film was analyzed using a Kodak Image Station 440. Quantitation was based on comparisons to internal standard DNA containing a known amount of AP sites.

### Fluctuation Analysis of Frameshift Mutations

The plasmids used in the study were described previously [12]. Lithium acetate method (Geitz kit, Genomics One International Inc. Buffalo, NY) was used to introduce YEpAPN1, pYES2.0, and pYES-MAG plasmids into isogenic haploid *apn1* null E133 and E134 yeast strains [22]. The lys2:*InsE* inserts inactivate the *LYS2* gene with 12 and 14 nucleotide runs of A, respectively, resulting in a Lys^−^ phenotype. E133 reverts to Lys^+^ solely by a +1 frameshift, whereas E134 reverts by a −1 frameshift mutation. *S. cerevisiae* 3MeA DNA glycosylase (MAG) was expressed in yeast when galactose, at final concentration of 2%, was added to SC drop-out media lacking uracil (Q-BioGene, Carlsbad, CA). The *S. cerevisiae* AP endonuclease 1 (APN1) was expressed from the YEpAPN1 plasmid maintained in culture by excluding leucine from the SC drop-out media. Transformant cultures were grown overnight in 2% synthetic glucose media without uracil for pYES2.0 and pYES-MAG or without uracil and leucine when YEpAPN1 was additionally included in combination with the above plasmids. When cultures reached saturation, they were diluted to a density of ∼4,000 cells/ml in 2% galactose SC media and divided into ten parallel 10 ml cultures. After incubation at 30°C for ∼4 days, cells were harvested, washed, and resuspended in 1 ml water. Dilutions were spread on SC–uracil or SC–uracil–leucine plates, respectively, to determine the average number of cells per culture for three randomly chosen samples per each condition. Duplicates of 0.1 ml aliquots of each culture were spread on SC +galactose plates lacking lysine to select for Lys^+^ revertants. After ∼4–5 day incubation at 30°C, colonies were counted. The median number of revertants per 10^8 ^cells was obtained and is expressed as mean±standard deviation from at least 3 independent experiments.

### RNA extraction and Microarray Analysis

Total RNA was isolated using hot phenol extraction and hybridized to Affymetrix® (Santa Clara, CA) Yeast Genome S98 arrays in biological triplicate. RNA clean-up, cRNA labeling and fragmentation, array hybridization and staining were done as described in the Affymetrix® eukaryotic labeling protocol (Santa Clara, CA). Processed microarrays were scanned and raw image intensity data files were processed and normalized using robust multi-array average (RMA) software [23]. Statistical significance of expression was determined for each gene using triplicate arrays for each experimental group of BER relative to the wild type strain (ANOVA, p<0.05; Fold change ≥1.5, ≤−1.5). All microarray data have been submitted to the Gene Expression Omnibus, accession number GSE9295 (www.ncbi.nlm.nih.gov/geo/).

### Network Analysis, Gene Ontology and ESR Enrichment

Network analysis was carried out using the Cytoscape software [24]. Transcriptionally modulated ORFs were linked into the large-scale probabilistic network [17]. Statistical evaluation of co-regulated interacting groups of genes was carried out through Gene Ontology Enrichment Analysis within the Functional Specification Database [25]. To classify transcripts as activated or repressed across the BER experiments a mean expression value was calculated and queried for Gene Ontology enrichment as above. Co-regulated yeast ORFs were classified according to the Gene Ontology Biological Process and the hypergeometric distribution used to assess enrichment of a particular gene category. Statistical significance of enrichment of environmental stress genes was calculated through the Fisher's Exact Test.

## Supporting Information

Figure S1 BER modulated transcriptional networks in *S. cerevisiae*. A) The transcripts modulated under BER intermediate accumulation were integrated with their gene products and overlayed on all known protein-protein interactions in yeast (4,681 nodes and 34,000 edges). Of the BER modulated ORFs, 501 ORFs were identified in the database of known protein-protein interactions in the large interacting network (indicated in red). B) Analysis requiring that all interacting nodes are transcriptionally modulated by BER intermediate accumulation results in the identification of five subnetworks (1–5) significantly enriched for GO biological processes.
(7.69 MB EPS)

Dataset S1 List of 633 ORFs modulated by BER imbalance. ORFs modulated in at least one condition of base excision repair imbalance in yeast were identified using differential expression testing.
(0.21 MB XLS)
